# Novel missense mutations in PTCHD1 alter its plasma membrane subcellular localization and cause intellectual disability and autism spectrum disorder

**DOI:** 10.1002/humu.24208

**Published:** 2021-05-03

**Authors:** Judith Halewa, Sylviane Marouillat, Manon Dixneuf, Rose‐Anne Thépault, Dévina C. Ung, Nicolas Chatron, Bénédicte Gérard, Jamal Ghoumid, Gaëtan Lesca, Marianne Till, Thomas Smol, Nathalie Couque, Lyse Ruaud, Valérie Chune, Sarah Grotto, Alain Verloes, Marie‐Laure Vuillaume, Annick Toutain, Martine Raynaud, Frédéric Laumonnier

**Affiliations:** ^1^ UMR1253, iBrain, INSERM University of Tours Tours France; ^2^ Department of Genetics Hospices Civils de Lyon Lyon France; ^3^ Institut NeuroMyoGène, CNRS UMR‐5310, INSERM U‐1217, Univ Lyon Université Claude Bernard Lyon 1 Lyon France; ^4^ Laboratoire de diagnostic génétique, Institut de Génétique Médicale d'Alsace Hôpitaux Universitaires de Strasbourg Strasbourg France; ^5^ EA7364 RADEME, Clinique de Génétique Guy Fontaine, Université de Lille CHU de Lille Lille France; ^6^ EA7364 RADEME, Institut de Génétique Médicale, Université de Lille CHU de Lille Lille France; ^7^ Department of Genetics APHP‐Robert Debré University Hospital Paris France; ^8^ INSERM, UMR1141, Denis Diderot School of Medicine Paris University Paris France; ^9^ Service de Génétique Centre hospitalier régional universitaire de Tours Tours France

**Keywords:** autism spectrum disorder, functional analyses, in vitro cellular models, intellectual disability, missense variants, *PTCHD1* (patched domain containing 1) gene, subcellular localization

## Abstract

The X‐linked *PTCHD1* gene, encoding a synaptic membrane protein, has been involved in neurodevelopmental disorders with the description of deleterious genomic microdeletions or truncating coding mutations. Missense variants were also identified, however, without any functional evidence supporting their pathogenicity level. We investigated 13 missense variants of PTCHD1, including eight previously described (c.152G>A,p.(Ser51Asn); c.217C>T,p.(Leu73Phe); c.517A>G,p.(Ile173Val); c.542A>C,p.(Lys181Thr); c.583G>A,p.(Val195Ile); c.1076A>G,p.(His359Arg); c.1409C>A,p.(Ala470Asp); c.1436A>G,p.(Glu479Gly)), and five novel ones (c.95C>T,p.(Pro32Leu); c.95C>G,p.(Pro32Arg); c.638A>G,p.(Tyr213Cys); c.898G>C,p.(Gly300Arg); c.928G>C,p.(Ala310Pro)) identified in male patients with intellectual disability (ID) and/or autism spectrum disorder (ASD). Interestingly, several of these variants involve amino acids localized in structural domains such as transmembrane segments. To evaluate their potentially deleterious impact on PTCHD1 protein function, we performed in vitro overexpression experiments of the wild‐type and mutated forms of PTCHD1‐GFP in HEK 293T and in Neuro‐2a cell lines as well as in mouse hippocampal primary neuronal cultures. We found that six variants impaired the expression level of the PTCHD1 protein, and were retained in the endoplasmic reticulum suggesting abnormal protein folding. Our functional analyses thus provided evidence of the pathogenic impact of missense variants in PTCHD1, which reinforces the involvement of the *PTCHD1* gene in ID and in ASD.

## Graphical abstract

Thirteen missense variants in the PTCHD1 gene associated with X‐linked neurodevelopmental disorder were investigated to address their potentially deleterious impact. Their overexpression in various cell lines (HEK293T, Neuro‐2a) and in primary neuronal cultures revealed abnormal protein stability and impaired subcellular localization, thus providing further evidence for the significant impact of PTCHD1 genetic alterations in intellectual disability and in autism spectrum disorder.

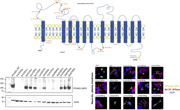

## 1 BACKGROUND

From the last few decades, the molecular study of neurodevelopmental disorders revealed the involvement of numerous genes essential in neuronal developmental processes like morphogenesis or neuronal plasticity and synaptogenesis (Bourgeron, 2015; Gilman et al., 2011; Krishnan et al., 2016; Laumonnier et al., 2007; Moyses‐Oliveira et al., 2020; Parenti et al., 2020). Among them, microdeletions and mutations in the X‐chromosomal *PTCHD1 (patched domain containing 1)* gene were described in patients with autism spectrum disorders (ASDs) and/or intellectual disability (ID; Chaudhry et al., 2015; Filges et al., 2011; Marshall et al., 2008; Noor et al., 2010). More precisely, *PTCHD1* gene disruption or truncation causes a recessive X‐linked non‐syndromic neurodevelopmental disorder characterized by variable features of ID, ASD, global developmental delay in their childhood with also infantile hypotonia (especially hypotonic face features), motor incoordination, and sometimes dysmorphic features (Pinto et al., 2010). Some patients display behavioral or psychiatric issues, including attention deficit with or without hyperactivity (ADHD), sleep disruptions, aggressive, or impulsive behavior (Chaudhry et al., 2015). No evidence for a recognizable and significant pattern of congenital anomalies or serious medical co‐morbidities in association with disruptions or deletions of this gene was highlighted.

*PTCHD1* encodes a membrane protein located in GABAergic neurons (Wells et al., 2016) and in glutamatergic neurons (Tora et al., 2017), with 12 predicted transmembrane domains, two large extracellular loops, and intracellular amino and carboxy‐terminal tails. Recent studies highlighted that PTCHD1 can bind postsynaptic scaffold proteins PSD95 and DLG3 (SAP102) via a PDZ binding domain motif at the end of the C terminal tail (Tora et al., 2017; Ung et al., 2018), as well as retromer complex proteins SNX27, and also VPS26B and VPS35 but independently from the PDZ‐domain‐binding motif (Tora et al., 2017). PTCHD1 is also predicted to be a receptor, homologous to PTCH1, which is involved in the Sonic Hedgehog (SHH) pathway, however, PTCHD1 does not include the SHH ligand‐binding site motif which is present in PTCH1 and has been found unable to modulate the SHH pathway (Bosanac et al., 2009; Ung et al., 2018).

Single nucleotide variants (SNVs), including missense, nonsense or truncating mutation in PTCHD1 have been highlighted in patients with NDD. Strikingly, little is known about the pathogenic evaluation of PTCHD1 missense variants using biological in vitro experiments, which leads to significant issues in genetic medical diagnosis. We established a functional analysis to investigate 13 missense variants using in vitro non‐neuronal (HEK 293T) and neuronal (Neuro‐2a and primary neurons) cellular models. Here, we report findings showing differential impacts of PTCHD1 variants on the cell membrane localization pattern, as well as protein expression levels. Specifically, variants located in structural domains such as transmembrane segments abolished the membrane expression of PTCHD1, which was retained in the endoplasmic reticulum. Besides the increasing number of pathogenic microdeletions and truncating mutations of PTCHD1 causing ASD and ID, our data provide further evidence of the role of PTCHD1 in neurodevelopmental disorders, with the growing contribution of missense mutations leading to loss‐of‐function consequences.

## 2 METHODS

### 2.1 Patients and identification/follow‐up of missense variants in *PTCHD1*

The genetic investigations regarding the patients with the five novel PTCHD1 variants were approved by the local Institutional Review Boards (Lille, Lyon, Paris, Strasbourg), and written informed consent was obtained from the patients' parents, including explicit permission to share clinical and identifying information. The molecular analyses were performed through genetic diagnosis protocols using whole‐exome strategies on the affected boy and co‐segregation processed on parents' genomic DNA when available. The collection of these variants was possible through the French National Genetics Network on Intellectual Disability. The eight published missense variants have been reported in the SFARI gene database (https://gene.sfari.org).

### 2.2 Pathogenicity prediction of the variants

The *PTCHD1* variants are named according to the isoform Refseq NM_173495.3 which encodes a protein of 888 amino acids (Refseq NP_775766.2). The potential effects of missense variants were predicted using SIFT (Kumar et al., 2009; https://sift.bii.a-star.edu.sg/), Polyphen‐2 (Adzhubei et al., 2010; http://genetics.bwh.harvard.edu/pph2/), and UMD Predictor (Salgado et al., 2016; http://umd-predictor.eu/). As SIFT is a tool relying on sequence homology between species, the protein sequence was taken from all species referred to on Uniprot (https://www.uniprot.org/uniprot/), corresponding to 816 complete or partial PTCHD1 orthologues. Protein sequences were aligned with Clustal Omega (Sievers et al., 2011) on Uniprot. In SIFT, the human PTCHD1 protein sequence (Refseq NP_775766.2) was entered as the query sequence, the others as related sequences in SIFT Aligned Sequences tool, with the FASTA file of aligned sequences generated by Uniprot after the alignment. In UMD‐Predictor, “single analysis” was performed on the human *PTCHD1* transcript ENST00000379361 from Ensembl (https://www.ensembl.org/). The presence of the different variants in the general population was assessed using gnomAD database (http://gnomad.broadinstitute.org/, v2.1.1), including both total and “non‐neuro” data sets.

### 2.3 PTCHD1 expression plasmid and site‐directed mutagenesis

The sequence of full‐length coding *Ptchd1* mouse cDNA was amplified from the IMAGE cDNA clone 40095445 (GenBank accession number BC116312; Source BioSience) and cloned in pAcGFP1‐N vectors using In‐Fusion cloning strategy (Catalog no. 632501, Clontech) to generate PTCHD1‐GFP with GFP tag at the C‐terminal end (previously described in Ung et al. (2018)). Importantly, the murine PTCHD1 protein shares the same amino acids length (888) with 98% of sequence identity between murine and human PTCHD1 proteins. The mutant PTCHD1‐GFP plasmids were generated using the Q5® Site‐Directed Mutagenesis Kit (New England Biolabs) according to the manufacturer's recommendations. The primer sequences for each mutagenesis are indicated in Table S1. The efficiency of the site‐directed mutagenesis was verified by Sanger Sequencing.

### 2.4 HEK 293T and Neuro‐2a cell lines culture

HEK 293T cell lines (ATCC, CRL‐3216) were cultured with DMEM (Gibco, ref 31966047) medium, with 1% of sodium pyruvate solution (100 mM, S8636, Sigma‐Aldrich®) and 1% MEM non‐essential amino acids solution (M7145, Sigma‐Aldrich®) and 10% of fetal bovine serum (FBS; Eurobio, CVFSVF06‐01). Neuro‐2a (N2A) cell lines (ATCC, CCL‐131) were cultured with EMEM medium (ATCC, 30‐2003) and 10% of FBS. Both cell lines were subcultured twice a week by trypsinization (trypsin 0.25%, Gibco, 25200056).

### 2.5 Primary neuronal cultures

All mouse experiments performed at the University of Tours/INSERM were approved by the French Ministry of Research (Project authorization number 01456.03). Brain tissues were dissected from embryonic Day 17.5 C57BL/6J WT mouse embryos (Janvier Labs) on cold DPBS with 1% of penicillin‐streptomycin (PS; Thermo Fisher Scientific), and the hippocampi were incubated with papain (10 U/ml; Worthington) for 22 min at 37°C, then mechanically dissociated in DMEM:F12 (Gibco, 31331093) with 10% of FBS (Eurobio, CVFSVF06‐01) and centrifuged at 250*g* for 5 min. The cellular pellet was resuspended in Neurobasal Plus (Gibco, A3582901) supplemented with B27 Plus (Gibco A35828‐01) 1X and l‐glutamine (0.5 mM, Gibco, 25030149). Dissociated neurons were seeded on glass coverslips coated with Poly‐d‐lysine (Sigma, P7280) and laminin (4 µg/ml; Gibco, 23017‐015) with a density of 400 cells per mm². Half of the medium was changed twice a week.

### 2.6 Transfection

Hippocampal neurons (at 11th day of culture in vitro), HEK 293T, and Neuro‐2a cell lines were transfected with a plasmid construct (WT or mutated forms of PTCHD1‐GFP) using Lipofectamine 2000 (Thermo Fisher Scientific, 11668027) with 1 µg of DNA/2 µl of L2000 diluted in Neurobasal medium. The mix was added to cells and they were incubated for 4 h (37°C, 5% CO_2_), then the transfection medium was replaced with a mix 1:1 of new complete culture medium and previously removed medium (conditioned medium) for neurons or only new medium for cell lines. Microscopy analyses were carried out 48 h after transfection.

### 2.7 Gene expression study by qPCR

HEK 293T cells were transfected with pAc‐PTCHD1‐GFP different plasmids using lipofectamine 2000 as described above. Two days after transfection total RNA was extracted from HEK 293T samples with TRIzol and then purified using a DirectZol Kit (Ozyme). About 1 µg of RNA was used to generate cDNA using a PrimeScript RT Reagent Kit (Takara, RR037B). PCR amplification of *Ptchd1* cDNA was performed with a forward primer located in exon 1 is 5ʹ‐GCCAACATGCTAGACCAACA‐3ʹ, and the reverse primer located in exon 2 is 5ʹ‐CCCGAGCATTCTTTAGCTCTT‐3ʹ. The human *GAPDH* cDNA was used as a reference gene with a forward primer: (5ʹ‐CTGCACCACCAACTGCTTAG‐3ʹ) and a reverse primer (5ʹ‐GTCTTCTGGGTGGCAGTGAT‐3ʹ). PCR analyses were done in duplicates using SYBR Green Takyon Kit (Eurogentec, UF‐NSMT‐B0701) and a LightCycler 480 (Roche) on total cDNAs obtained from four independent cultures. Data were normalized to the *GAPDH* gene and to the pAc‐PTCHD1‐GFP WT control according to the 2^−∆∆Cp^ method. Data statistical analysis was carried out with GraphPad Prism 8, using a Kruskal–Wallis test with Dunn's multiple comparisons from at least four independent experiments.

### 2.8 Western blot

Two days posttransfection, cells were washed two times with cold PBS then were lysed using RIPA buffer (Pierce) with protease inhibitors cocktails for 15 min on ice. After incubation, the lysates were centrifugated at 15,000*g* for 10 min. The supernatants were collected and total protein lysates were quantified. A total of 30 µg of non‐denatured proteins were processed for SDS‐PAGE at 180V during 1 h using mini‐PROTEAN precast gels 4%–20% TGX stain‐free (456‐8094, Bio‐Rad). SDS‐PAGE gels were blotted on PVDF membrane using TransBlot Turbo (Bio‐Rad) followed by blocking the membrane for 1 h with 5% milk diluted in TBS‐Tween (0.05% tween 20). Primary antibodies for PTCHD1‐GFP (1/2000, 632592, TakaraBio) and actin (1:100,000, A3854, Sigma‐Aldrich) were incubated overnight at 4°C in 5% milk in TBST buffer. After three washes (10 min) with TBST, membranes were incubated with corresponding HRP‐conjugated secondary antibodies (Goat anti‐rabbit 1/2500, W4018, Promega) for 45 min at room temperature. Proteins were detected after 1‐min incubation for Actin blot and 5‐min incubation for PTCHD1‐GFP blot with Clarity western ECL Kit (1705061, Bio‐Rad), and the membranes were visualized in a Chemidoc Touch imaging system (Bio‐Rad). Immunoblots were analyzed by Chemidoc Touch (Bio‐Rad) by calculating the volume of each band for the same area. The bands' intensity was normalized to Actin. Data statistical analysis was carried out using GraphPad Prism 8 and using the Kruska–Wallis test with Dunn's multiple comparisons test.

### 2.9 Immunocytochemistry

Cells cultured on coverslips were fixed in a solution of paraformaldehyde 4%/sucrose 4%, 2–3 days after transfection. They were incubated for 20 min at room temperature with fixation solution, then conserved in DPBS at 4°C until immunofluorescence staining preparation. Fixed cells were blocked and permeabilized by a DPBS solution with 10% of Donkey serum (DS; Sigma‐Aldrich) and 0.2% Triton X‐100 (Sigma‐Aldrich) for 1 h at room temperature (RT). They were incubated in a similar solution (DPBS, 0.2% Triton X‐100, 3% DS) containing primary antibodies: GFP rabbit Polyclonal Antibody (1/200, TakaraBio, 632592), mouse monoclonal anti‐GFP (1/200, Sigma‐Aldrich, 11814460001), PSD‐95 mouse Monoclonal Antibody 7E3‐1B8 (1/200, Thermo Fisher Scientific, MA1‐046), PSMB5 rabbit Polyclonal Antibody, (1/200, Thermo Fisher Scientific, PA1‐977) Calnexin mouse monoclonal antibody (1/500, Thermo Fisher Scientific, MA3‐027), anti‐Sodium Potassium ATPase rabbit monoclonal antibody (1/500, Abcam, ab76020), β3‐tubulin chicken polyclonal antibody (1/500, Synaptic Systems, 302306) for 1 h at RT. After three washes in DPBS for 10 min each, the cells were incubated for 45 min at RT with polyclonal secondary antibodies coupled to fluorophores, diluted in the same solution (DPBS, 0.2% Triton X‐100, 3% DS): FP‐488 Donkey anti‐mouse IgG antibody (1/300), FP‐488 Donkey anti‐rabbit IgG antibody (1/300), FP‐546 Donkey anti‐mouse IgG antibody (1/300–1/500), FP‐546 Donkey anti‐rabbit IgG antibody (1/300) (Interchim) and CF™405 Goat anti‐chicken IgG antibody (1/500, Sigma‐Aldrich, SAB4600466). The cells were mounted with the DAPI ProLong gold antifade solution (Thermo Fisher Scientific).

### 2.10 Image analysis

The cellular‐imaging study was performed using a confocal microscope Leica SP8 and the associated software Leica Application Suite X (LAS X). The White Light Laser (WLL; set at 546 nm) maximum intensity was limited at 70%, the 488 nm argon laser at 30%, and the 405 nm diode. Inner settings were performed depending on the emitted fluorescence from cells. Sequential acquisitions were made, and high‐resolution z stack images of cells were taken with the optical magnification of 6× 300 with optical section separation (z interval) of 0.6 µm. The Image colocalization Manders' coefficient (Aaron et al., 2018) was used to quantify PTCHD1‐GFP overlapping with each used co‐marker (calnexin, PSMB5, and Na^+^/K^+^ APTase) and was calculated via Fiji software (with automatic thresholds), on three consecutive stack image per condition. Data analysis was carried out using GraphPad Prism 8. We used a d'Agostino–Pearson Normality test followed by an Analysis of variance (ANOVA) Kruskal–Wallis test with Dunn's multiple comparisons test to compare the variants of PTCHD1 with WT PTCHD1, on at least three independent experiments.

## 3 RESULTS

### 3.1 Missense variants in PTCHD1 in individuals with a neurodevelopmental disorder

**Figure 1:**
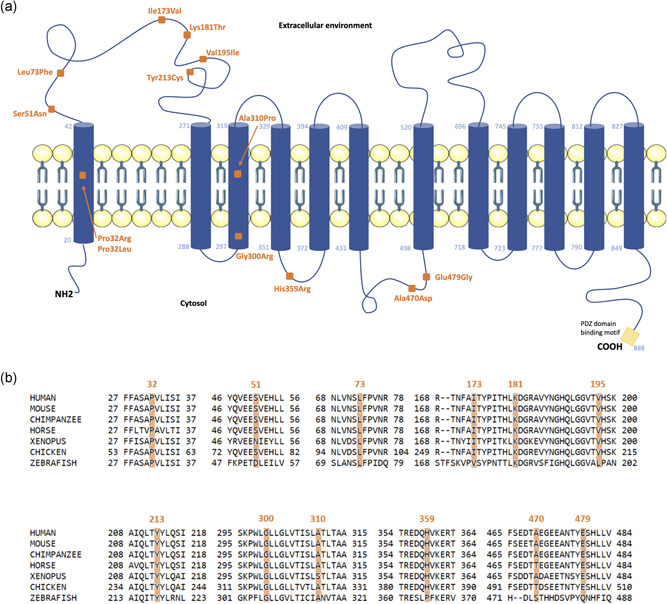
Schematic representation of the structural organization of PTCHD1 protein and localization of the variants. (a) The predicted secondary structure of PTCHD1 protein (NP_775766.2, 888 amino acids) has been generated using TMHMM server 2.0 (http://www.cbs.dtu.dk/services/TMHMM/), revealing the 12 transmembrane domains (their respective boundaries are indicated by the amino acid number) embedded in the plasma membrane structure, the intracellular loops, N‐terminal, and C‐terminal ends, as well as the two extracellular loops. The missense variants are positioned with orange‐colored squares, transmembrane (TM) domains by blue cylinders, and the PDZ‐domain‐binding motif (ITTV) is represented at the end of the C‐terminal tail by a yellow box. (b) Clustal Omega protein sequences alignment of PTCHD1 orthologs of regions surrounding the amino acids replaced by the missense variants (highlighted in orange). The PTCHD1 orthologs Uniprot RefSeq are: Mouse (Q14B62), chimpanzee (H2QYE5), horse (F6XSF0), Xenopus (A0A6I8SSY4), and zebrafish (Q5RIV7)

Based on the literature and on the SFARI Gene database (https://gene.sfari.org/database/human-gene/PTCHD1#variants-tab), we first collected eight missense variants. The p.(Leu73Phe), p.(Ile173Val), p.(Val195Ile), p.(His359Arg), p.(Ala470Asp), and p.(Glu479Gly) mutations were described in patients with ASD, ID, or other NDD (Noor et al., 2010), the p.(Ser51Asn) variant was reported in a patient with ID (Torrico et al., 2015), and the p.(Lys181Thr) variant was identified by whole‐exome sequencing in a patient with frontal lissencephalic cortical dysplasia and seizures (Karaca et al., 2015; Table 1). As no functional validation was performed on these variants, the authors did not conclude about their pathogenicity degree. The five remaining, novel variants included in our study were identified in the French National Genetics Network on Intellectual Disability for genetic molecular diagnosis purposes. The p.Pro32Arg mutation was found in a patient with ID and inherited from his mother; the p.(Pro32Leu) was identified in a patient with NDD and was also present in an uncle without the description of NDD. We also collected patients with NDD and carrying the p.(Gly300Arg), p.(Tyr213Cys) variants. Interestingly, we detected a de novo p.(Ala310Pro) variant in a patient with ID. None of these SNVs were found in the gnomAD database, with the exception of the p.(Leu73Phe), p.(Ile173Val), and p.(Val195Ile) changes detected in 5, 34, and in 1 male individual, respectively (Table 1). Based on the predicted secondary structure of the PTCHD1 protein, we were able to locate the missense variants in distinct structural domains, such as transmembrane segments (Proline 32 in the first (TM) domain, Glycine 300, and Alanine 310 in the third TM domain), the extracellular loop (Serine 51, Leucine 73, Isoleucine 173, Lysine 181, Valine 195, and Tyrosine 213), or intracellular loops (Histidine 359, Alanine 470, and Glutamate 479; Figure 1a).

**Table 1:** Clinical, molecular, in silico pathogenicity prediction details, and functional analyses on the 13 missense PTCHD1 variants included in the study

PTCHD1 variant	*PTCHD1* exon	Localization in *PTCHD1* (NM_173495.3)	Protein domain	Neurodevelopment phenotype	Inheritance	Other genetic findings	Hemizygotes in gnomAD v2.1.1 (total, non‐neuro) vs. total number of alleles screened	Pathogenicity prediction scores			Plasma Membrane localization	References
								SIFT	Polyphen‐2 (HumDiv/HumVar)	UMD predictor		
p.Pro32Leu	Exon 1	c.95C>T	1st TM	NDD	Maternal	Detected in one uncle without NDD	None	Not tolerated	Probably damaging	Pathogenic	Impaired	Novel (Lyon)
p.Pro32Arg	Exon 1	c.95C>G	1st TM	ID, ASD, congenital ataxia (normal brain MRI at 2 years old)	Maternal	n/a	None	Not tolerated	Probably damaging	Pathogenic	Impaired	Novel (Lille)
p.Ser51Asn	Exon 1	c.152G>A	1st EC loop	ID	n/a	n/a	None	Tolerated	Benign	Polymorphism	Yes	Torrico et al. (2015)
p.Leu73Phe	Exon 1	c.217C>T	1st EC loop	ASD	Maternal	n/a	Total: 5/182,819	Not tolerated	Benign	Polymorphism	Yes	Noor et al. (2010)
							Non‐neuro: 5/151,285					
p.Ile173Val	Exon 2	c.517A>G	1st EC loop	Proband 1: ASD	Maternal	De novo del. including *DPYD* in proband 2	Total: 34/205,280	Tolerated	Benign	Polymorphism	Yes	Noor et al. (2010)
				Proband 2: ASD, ID hyperactivity, weak motor coordination			Non‐neuro: 31/166,801					
p.Lys181Thr	Exon 2	c.542A>C	1st EC loop	Cortical dysplasia, frontal lissencephaly	Maternal	n/a	None	Tolerated	Benign	Pathogenic	Impaired	Karaca et al. (2015)
p.Val195Ile	Exon 2	c.583G>A	1st EC loop	ASD, speech disorder	Maternal	De novo del. of *DPP6*	Total: 1/183,367	Tolerated	Probably damaging/Benign	Polymorphism	Yes	Noor et al. (2010)
							Non‐neuro: 1/151,846					
p.Tyr213Cys	Exon 2	c.638A>G	1st EC loop	NDD	Maternal	n/a	None	Not tolerated	Probably damaging	Pathogenic	Impaired	Novel (Paris/Strasbourg)
p.Gly300Arg	Exon 2	c.898G>C	3rd TM	NDD	maternal	n/a	None	Not tolerated	Probably damaging	Pathogenic	Impaired	Novel (Lille)
p.Ala310Pro	Exon 2	c.928G>C	3rd TM	NDD	De novo	De novo 16p11.2 del.	None	Not tolerated	Probably damaging	Pathogenic	Impaired	Novel (Paris)
p.His359Arg	Exon 3	c.1076A>G	IC loop between 4th and 5th TM	Severe ID	Maternal	+2 Kb del. involving last exon of *SLC16A2*	None	Not tolerated	Benign	Pathogenic	Yes	Noor et al. (2010)
p.Ala470Asp	Exon 3	c.1409C>A	IC loop between 6th and 7th TM	Moderate ID	Maternal	n/a	None	Tolerated	Benign	Polymorphism	Yes	Noor et al. (2010)
p.Glu479Gly	Exon 3	c.1436A>G	IC loop between 6th and 7th TM	ASD	Maternal	n/a	None	Not tolerated	Probably damaging	Pathogenic	Yes	Noor et al. (2010)

Abbreviations: ASD, autism spectrum disorder; del., deletion; EC, extracellular; IC, intracellular; ID, intellectual disability; NDD, neurodevelopmental disorder; n/a, not available; TM, transmembrane domain.

### 3.2 The PTCHD1 protein is relatively intolerant to missense changes

We reviewed all the missense variants present in the general population using the full gnomAD data set (v2.1.1, 141,456 samples). Almost 30% fewer missense variants were described when compared to what would be expected (238/342), which suggests that the *PTCHD1* gene would be relatively intolerant to missense changes (indicated *Z*‐score = 2). More specifically, 234 missense variants are reported in the gnomAD “non‐neuro” cohort (114,704 samples), that is, in individuals not ascertained with a neurological or psychiatric condition. The prediction of the deleterious effect of the 13 missense changes in PTCHD1 protein showed a wide range of scores, using SIFT, Polyphen‐2, and UMD‐Predictor software (Table 1). Although all missense variants involve amino acids located in the conserved region of PTCHD1 orthologs within vertebrates (Figure 1b), only six variants were unanimously predicted potentially damaging, including the p.(Pro32Arg), p.(Pro32Leu), p.(Tyr213Cys), p.(Gly300Arg), p.(Ala310Pro), and p.(Glu479Gly). Consequently, without performing dedicated functional studies to assess the impact of the variants on subcellular localization of PTCHD1 at the plasma membrane, it would be difficult to interpret the pathogenicity level of the candidate variants found in patients with NDD only by in silico predictions.

### 3.3 Missense mutations impair PTCHD1 protein expression level in transfected HEK293 cells

**Figure 2:**
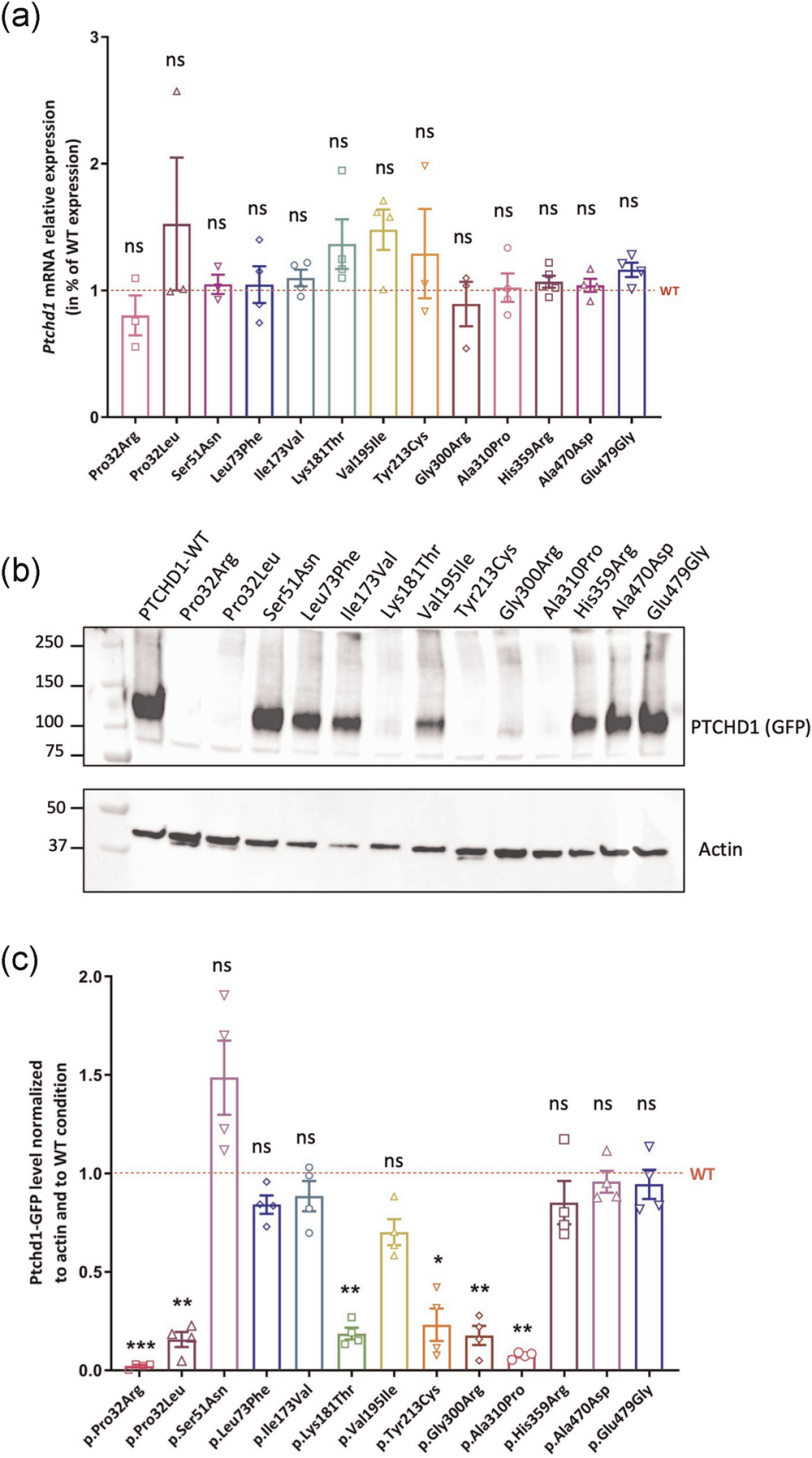
Expression analysis of the GFP‐tagged wild‐type and variant PTCHD1 mRNAs and proteins in transfected HEK293T cells. (a) Expression analysis of *Ptchd1* mRNAs in HEK293T cells transfected with PTCHD1‐GFP expression plasmids during 48 h. Quantification of mRNA expression levels was measured by RT‐qPCR using the reference gene *GAPDH* on total RNA lysates extracted from three to four independent transfected cultures. Data were normalized to *GAPDH* and to PTCHD1‐WT condition. Analysis of variance (ANOVA) Kruskal–Wallis tests and Dunn's multiple comparisons test were done to compare the expression of variant PTCHD1 with WT mRNA expression. (b) Expression analysis of PTCHD1‐GFP WT and variant proteins in transiently transfected HEK 293T cells. The PTCHD1‐GFP protein (130 kDa) expression was detected by SDS‐PAGE and immunoblotting using an anti‐GFP antibody and the actin protein was used as a reference. (c) Expression analysis of PTCHD1‐GFP proteins overexpressed in HEK293T cells 48 h posttransfection. Quantifications were performed using expression of actin protein on four independent transfected cultures, and measurements were normalized to actin and WT conditions. ANOVA Kruskal–Wallis tests and Dunn's multiple comparisons test were done to compare the expression of variant PTCHD1 with WT protein. ns, not significant; SDS‐PAGE, Sodium Dodecyl Sulfate‐polyacrylamide gel electrophoresis; SEM, standard error of the mean; WT, wild‐type. *p < .05; **p < .01; ***p = .001; error bars represent SEM

To better determine the pathogenicity degree of the missense variants, we performed in vitro functional analyses using transfection experiments of WT and variants PTCHD1‐GFP expression plasmids in various cell lines, that is, HEK 293T (human), Neuro‐2a (mouse) cell lines, and mouse embryonic primary hippocampal neuronal cultures, thus providing various morphological details depending on the nonneuronal or differentiated neuronal cellular type. *Ptchd1* mRNA and protein expression levels were analyzed in HEK 293T cell lines, for which we did not detect any expression of an endogenous *PTCHD1* mRNA or protein (data not shown). As shown in Figure 2a, we confirmed that all Ptchd1‐GFP mRNAs were overexpressed at similar, not statistically different levels, whereas strong decreases were observed by Western blot analysis in the level of PTCHD1 variant proteins Pro32Arg, Pro32Leu, Lys181Thr, Tyr213Cys, Gly300Arg, and Ala310Pro, when compared to WT (Figure 2b,c). This suggests that the stability or expression of the PTCHD1 protein is likely affected, particularly by mutations involving amino acids located in transmembrane segments (Pro32, Gly300, and Ala310), or amino acids present in an extracellular loop and that might be crucial for its spatial organization. The remaining seven PTCHD1 variants were expressed at levels similar to PTCHD1‐GFP WT.

### 3.4 Missense variants impair PTCHD1 plasma membrane localization

We then performed subcellular localization studies of the WT and the 13 variant forms of PTCHD1 in HEK 293T, Neuro‐2a, and primary neuronal cultures. As seen in previous studies, the PTCHD1 protein is mostly expressed at the plasma membrane in neuronal and non‐neuronal cell lines, and also enriched in dendritic spines of neurons observed in primary cell culture (Noor et al., 2010; Tora et al., 2017; Ung et al., 2018).

**Figure 3:**
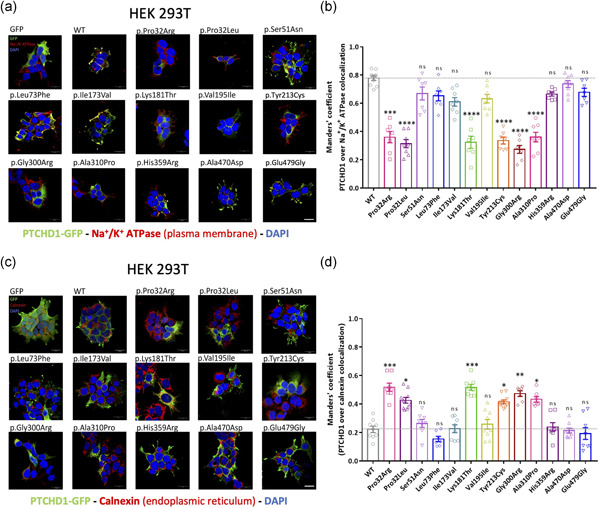
Subcellular localization of GFP‐tagged wild‐type and variant PTCHD1 proteins overexpressed in HEK293T cells. (a) Representative confocal microscopy images of HEK293T cells transfected with GFP, PTCHD1 expression plasmids, GFP‐tagged wild‐type (WT) or variants of PTCHD1, and stained with anti‐GFP antibody (PTCHD1‐GFP, green), anti‐Na^+^/K^+^ ATPase antibody (plasma membrane, red), and DAPI (nucleus, blue). n= 3 independent transfections. (b) Manders' coefficient of colocalization calculating the percentage of PTCHD1‐GFP WT or variants overlapping Na^+^/K^+^ ATPase (plasma membrane) staining. Kruskal–Wallis test with Dunn's multiple comparisons tests was made to compare each PTCHD1 variant to the WT. Normality of each condition was measured by the d'Agostino–Pearson normality test. For each condition, *n* = 7–10 images (each one indicated by one respective dot), from three independent transfections. (c) Representative confocal microscopy images of HEK293T cell lines transfected with GFP, PTCHD1 expression plasmids, GFP‐tagged wild‐type (WT) or variants of PTCHD1, and stained with anti‐GFP antibody (PTCHD1‐GFP, green), anti‐calnexin antibody (endoplasmic reticulum (ER), red) and DAPI (nucleus, blue). *n* = 3 independent transfections. (d) Manders' coefficient of colocalization calculating the percentage of Ptchd1‐GFP WT or variants overlapping calnexin (ER) staining. For each condition, *n* = 7–10 images (indicated by one respective dot), from three independent transfections. Scale bar, 20 µm; ns, not significant; SEM, standard error of the mean; *p < .05; **p < .01; ***p < .001; ****p < .0001; error bars represent SEM

**Figure 4:**
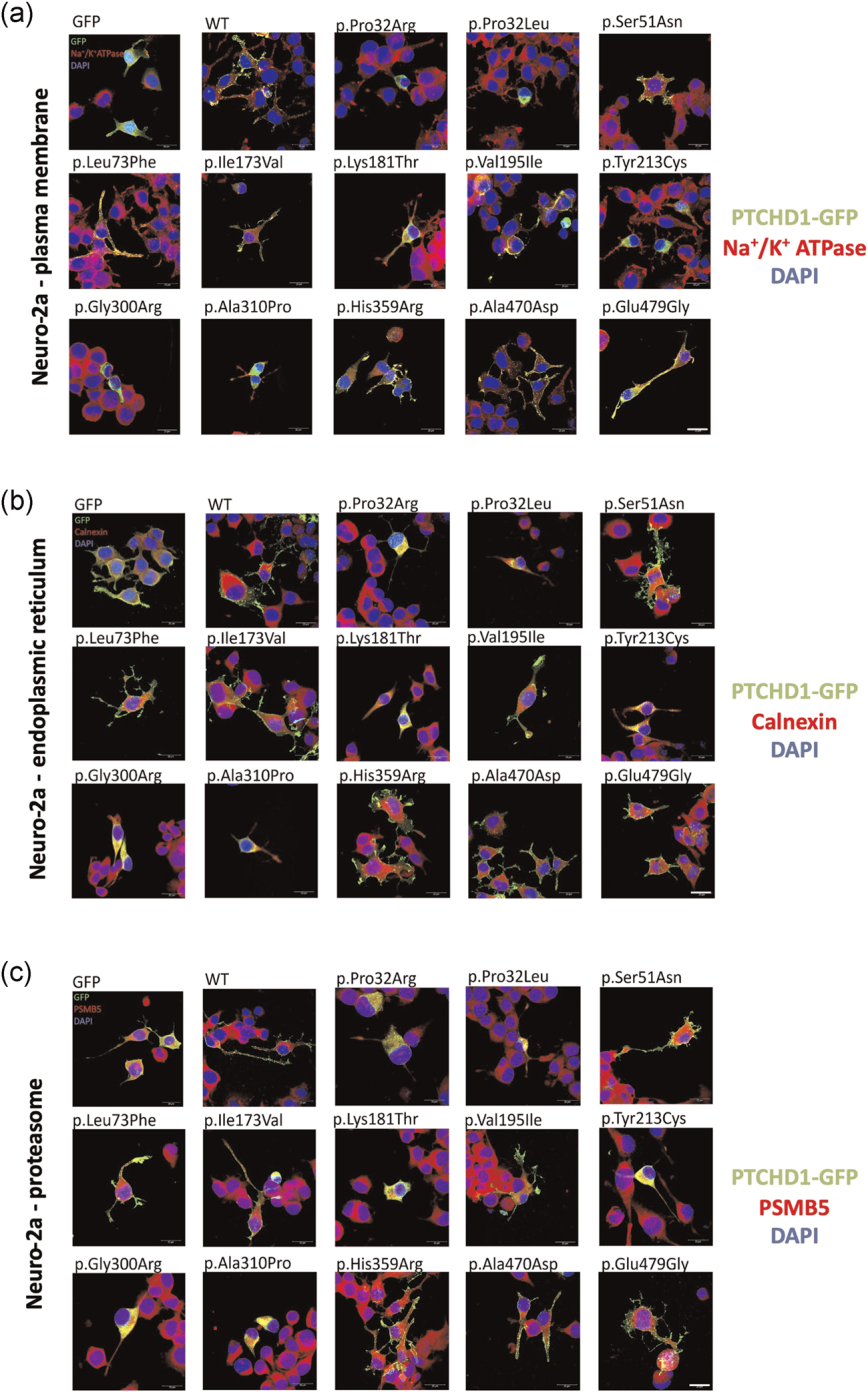
Subcellular localization of GFP‐tagged PTCHD1 proteins in transfected Neuro2a cells. Representative confocal microscopy images of immunofluorescence experiments in Neuro2a cells at 48 h posttransfection and fixation. The anti‐GFP antibody revealed the subcellular localization of Ptchd1‐GFP WT or variant forms (green fluorescence). The DAPI staining indicates the position of the nuclei. The subcellular compartments targeted in our analysis were: (a) the plasma membrane (Na^+^/K^+^ ATPase antibody, red), (b) the endoplasmic reticulum (Calnexin antibody, red), or (c) the proteasome 20S (PSMB5 antibody, red). *n* = 3 independent transfections, with a minimum of 6–8 images for each condition. Scale bar, 20 µm. WT, wild‐type

Immunostaining experiments revealed that the WT PTCHD1‐GFP and variant forms of PTCHD1 containing the Ser51Asn, Leu73Phe, Ile173Val, Val195Ile, His359Arg, Ala470Asp, or Glu479Gly variants are mostly and similarly localized at the plasma membrane in HEK 293T (Manders' coefficient mean value: 0.7785 for WT) and in Neuro‐2a transfected cells, as confirmed by co‐staining with the plasma membrane cell marker (Na^+^/K^+^ ATPase pump; Figure 3a,b). Conversely, we found a weak membrane localization for the PTCHD1 proteins with Pro32Arg, Pro32Leu, Lys181Thr, Tyr213Cys, Gly300Arg, and Ala310Pro variants (Figure 3a,b). These findings indicate that most of the PTCHD1 proteins with these six variants are not efficiently trafficked to the plasma membrane, which could be linked to abnormal folding of the extracellular domain (for the Lys181Thr and Tyr213Cys variants) or the transmembrane segments (For Pro32Arg, Pro32Leu, Gly300Arg, and Ala310Pro variants), leading to partial retention of the protein in the ER and subsequent degradation by the proteasome. We indeed confirmed the retention in the ER for the six variants using costaining with calnexin antibody (Figure 3c,d), as well as the colocalization with the PSMB5 antibody, a marker of proteasome P20S (Figure S1). Our comparative analysis in the mouse neuronal precursor Neuro‐2a cells transfected with the WT and variant forms of PTCHD1‐GFP confirmed our initial findings observed in HEK 293T cells (Figure 4).

Finally, these findings could explain the decreased protein expression levels found in our western blot analysis experiments.

**Figure 5:**
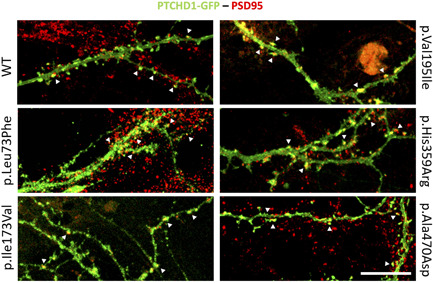
Synaptic localization of PTCHD1 variants in primary neuronal cultures. Mature hippocampal primary neuronal cultures were transiently transfected with PTCHD1‐GFP WT, p.Leu73Phe, p.Ile173Val, p.Val195Ile, p.His359Arg, and p.Ala470Asp variant constructs. The immunocytochemistry was performed using an anti‐GFP antibody (PTCHD1‐GFP, green) and an anti‐PSD95 antibody for labeling the dendritic spines (red). The synaptic overlapping signals (yellow dots) are indicated by white arrowheads. *n *= 2 independent transfections, with two neurons for each transfection. Scale bar, 15 µm. WT, wild‐type

Finally, we evaluated the subcellular localization of the PTCHD1 variants in primary cultures of mature hippocampal neurons which exhibit specific neuronal morphological features such as dendrites and dendritic spines. We confirmed that PTCHD1‐GFP WT was expressed in dendrites and dendritic spines, colocalizing with PSD95, a specific marker of the excitatory postsynaptic structures (Figure 5). We also showed that neurons overexpressing the PTCHD1‐GFP proteins carrying Ala470Asp, Leu73Phe, Ile173Val, His359Arg, and Val195Ile variants also displayed synaptic localization without any impairment in synaptic density (Figure 5). We could not detect any transfected neurons overexpressing the Pro32Arg, Pro32Leu, Ser51Asn, Lys181Thr, Tyr213Cys, Gly300Arg, Ala310Pro, and Glu479gly variants.

## 4 DISCUSSION

Mostly truncating mutations and genomic microdeletions of *PTCHD1* (either inherited or de novo) have been described in male individuals with X‐linked ID and ASD (Chaudhry et al., 2015; Deciphering Developmental Disorders Study, 2017; Filges et al., 2011; Gambin et al., 2017; Marshall et al., 2008; Noor et al., 2010; Pinto et al., 2010; Whibley et al., 2010). Furthermore, the available functional studies performed so far, in cellular and animal models, were all based on the absence of *PTCHD1* expression (Murakami et al., 2019; Tora et al., 2017; Ung et al., 2018; Wells et al., 2016). The analysis of the coding sequence of the *PTCHD1* gene in numerous cohorts of individuals with ID/ASD led to the description of few missense variants, however, without any relevant evidence supporting their pathogenicity.

We found that six missense mutations impair the membrane localization of PTCHD1, thus confirming that non‐synonymous missense variants in *PTCHD1* are probably associated with a neurodevelopmental disorder. We demonstrated that PTCHD1 proteins carrying Pro32Arg, Pro32Leu, Lys181Thr, Tyr213Cys, Gly300Arg, and Ala310Pro variants do not properly reach the plasma membrane in non‐neuronal or neuronal cells used for overexpression studies. This model suggests that mutated PTCHD1 proteins would not function as for the wild‐type protein in the developing human brain, leading to a possible loss‐of‐function consequence.

In view of these findings, we also found these six particular mutations induce a major decrease in PTCHD1 protein expression level. The associated pathophysiological mechanism is still speculative but would affect either the PTCHD1 sorting and trafficking toward plasma membrane, from the integration of PTCHD1 into ER to the vesicular transport, or the structural conformation of the protein, which will induce the proteasomal degradation process (Guna & Hegde, 2018; Ott & Lingappa, 2002). Other posttranslational processes such as abnormal glycosylation of the extracellular loops caused by variants located in these structural domains could be responsible, although we have not tested this hypothesis in our study. The Pro32Arg and Pro32Leu mutations are located in the first transmembrane (TM) domain and Gly300Arg and Ala310Pro are in the third TM domain. Consequently, the α helix structural organization of the TM domains would be affected with the replacement of hydrophobic residues (proline 32, glycine 300) by the charged arginine amino acid in the case of Pro32Arg and Gly300Arg. As PTCHD1 does not include a peptide signal sequence at its N‐terminal end, the first TM will contain the signal anchor sequence, which binds the signal recognition protein (SRP) during translation and SRP leads the ribosome‐nascent‐chain to the ER for translocation and membrane integration (Ott & Lingappa, 2002). The ineffective plasma membrane targeting of the PTCHD1 protein may be a direct clue supporting a pathological phenotype. Although disrupting variants in *PTCHD1* are highly penetrant (Chaudhry et al., 2015), we found that the Pro32Leu variant has also been detected in the patient's uncle who has not been diagnosed with NDD, suggesting a variable penetrance in this family, the presence of secondary genetic/nongenetic events that would compensate the impact of the PTCHD1 Pro32Leu variant, or the possibility that it might be unrelated to the disease.

We showed that the variants His359Arg, Ala470Asp, and Glu479Gly, which are located in intracellular loops, did not impair PTCHD1 membrane localization. Prediction pathogenicity data indicated that the His359Arg and Glu479Gly variants would be deleterious. We cannot exclude the possibility that these missense variants might impact potential protein interaction sites without altering PTCHD1 expression at the membrane, similarly to what was described for the C‐terminal intracellular tail (Ung et al., 2018), as well as for other synaptic proteins involved in NDD (Grant, 2019; Zhang‐James et al., 2019). The proband carrying the Glu479Gly mutation is diagnosed with high functioning autism, and the proband with the Ala470Asp mutation has a mild ID (Noor et al., 2010). The patient with the His359Arg mutation carries also a 2 kb deletion on Xq13.2 involving the last exon of *SLC16A2* (or monocarboxylate transporter 8, MCT8) gene involved in mild ID with hypotonia and in Allan–Herdon–Dudley syndrome (Remerand et al., 2019). Indeed Noor et al. (2010) noted that the patient has a severe ID with no ASD features and seizures, which is part of typical features of AHDS. Consequently, these mutations, in combination with other common or rare variants, may contribute to the neurodevelopmental phenotype, as previously suggested by Noor et al. (2010).

The ClinVar database (https://www.ncbi.nlm.nih.gov/clinvar/) reports 11 missense variants in *PTCHD1* involving amino acids included in the first extracellular loop (amino acids 43–271), whereas only two missense variants are referenced in the second extracellular loop (amino acids 521–695).

As classically undertaken in X‐linked disorders, the X‐inactivation status of the patient's mothers who transmitted their variant (although it is doubtful as to whether methylation status in mothers' lymphocyte DNA would bear any relation to that in the brain), as well as the segregation analyses in unaffected and/or affected relatives from different generations, would provide further arguments on the association of the candidate variant with the genetic disorder. This is however not always possible (i.e., de novo mutation or studies of trios cohorts), and the clinical validation of missense variants without functional evidence is still challenging to conclude about the pathogenicity degree, even with various prediction software. For example, we found that the variant Lys181Thr prevented membrane localization of PTCHD1 and caused ER retention, whereas it was predicted as benign by SIFT and Polyphen‐2 (but not with UMD predictor, Table 1). It is thus crucial to establish such experimental strategies to provide formal evidence for a relevant evaluation of missense candidate variants, as recently illustrated for the synaptic NLGN3 and NLGN4X proteins (Nguyen et al., 2020; Quartier et al., 2019), and to gain more knowledge on the structural organization and functional domains of the PTCHD1 protein, which is still an orphan receptor.

## CONFLICT OF INTERESTS

The authors declare that there are no conflict of interests.

## WEB RESOURCES

SFARI Gene database: https://gene.sfari.org

SIFT: https://sift.bii.a-star.edu.sg/

Polyphen‐2: http://genetics.bwh.harvard.edu/pph2/

UMD Predictor: http://umd-predictor.eu/

Uniprot: https://www.uniprot.org/uniprot/

Ensembl: https://www.ensembl.org/

GnomAD database: http://gnomad.broadinstitute.org/

## Supporting information

Supplementary information.

## Data Availability

The data that support the findings of this study are available from the corresponding author on reasonable request. The novel genetic (unpublished) variants of PTCHD1 have been submitted to the ClinVar database with the respective accession numbers (https://www.ncbi.nlm.nih.gov/clinvar/): SCV000890036.1 for p.(Pro32Leu), SUB954148 for p.(Pro32Arg), SCV001428357 for p.(Gly300Arg), VCV000988746.1 for p.(Tyr213Cys), and VCV000978387.1 for p.(Ala310Pro). The p.(Pro32Arg) variant is also described in DECIPHER (https://www.deciphergenomics.org) in subjects 284361 and 284363.

## References

[humu24208-bib-0001] Aaron, J. S., Taylor, A. B., & Chew, T. L. (2018). Image co‐localization – Co‐occurrence versus correlation. Journal of Cell Science, 131, jcs211847. 10.1242/jcs.211847 29439158

[humu24208-bib-0002] Adzhubei, I. A., Schmidt, S., Peshkin, L., Ramensky, V. E., Gerasimova, A., Bork, P., Kondrashov, A. S., & Sunyaev, S. R. (2010). A method and server for predicting damaging missense mutations. Nature Methods, 7, 248–249. 10.1038/nmeth0410-248 20354512 PMC2855889

[humu24208-bib-0003] Bosanac, I., Maun, H. R., Scales, S. J., Wen, X., Lingel, A., Bazan, J. F., de Sauvage, F. J., Hymowitz, S. G., & Lazarus, R. A. (2009). The structure of SHH in complex with HHIP reveals a recognition role for the Shh pseudo active site in signaling. Nature Structural and Molecular Biology, 16(7), 691–697. 10.1038/nsmb.1632 19561609

[humu24208-bib-0004] Bourgeron, T. (2015). From the genetic architecture to synaptic plasticity in autism spectrum disorder. Nature Reviews Neuroscience, 16, 551–563. 10.1038/nrn3992 26289574

[humu24208-bib-0005] Chaudhry, A., Noor, A., Degagne, B., Baker, K., Bok, L. A., Brady, A. F., Chitayat, D., Chung, B. H., Cytrynbaum, C., Dyment, D., Filges, I., Helm, B., Hutchison, H. T., Jeng, L. J., Laumonnier, F., Marshall, C. R., Menzel, M., Parkash, S., Parker, M. J., … Carter, M. T. (2015). Phenotypic spectrum associated with PTCHD1 deletions and truncating mutations includes intellectual disability and autism spectrum disorder. Clinical Genetics, 88(3), 224–233. 10.1111/cge.12482 25131214

[humu24208-bib-0006] Deciphering Developmental Disorders Study . (2017). Prevalence and architecture of de novo mutations in developmental disorders. Nature, 542, 433–438. 10.1038/nature21062 28135719 PMC6016744

[humu24208-bib-0007] Filges, I., Röthlisberger, B., Blattner, A., Boesch, N., Demougin, P., Wenzel, F., Huber, A. R., Heinimann, K., Weber, P., & Miny, P. (2011). Deletion in Xp22.11: PTCHD1 is a candidate gene for X‐linked intellectual disability with or without autism. Clinical Genetics, 79(1), 79–85. 10.1111/j.1399-0004.2010.01590.x 21091464

[humu24208-bib-0008] Gambin, T., Yuan, B., Bi, W., Liu, P., Rosenfeld, J. A., Coban‐Akdemir, Z., Pursley, A. N., Nagamani, S. C. S., Marom, R., Golla, S., Dengle, L., Petrie, H. G., Matalon, R., Emrick, L., Proud, M. B., Treadwell‐Deering, D., Chao, H. T., Koillinen, H., Brown, C., … Stankiewicz, P. (2017). Identification of novel candidate disease genes from de novo exonic copy number variants. Genome Medicine, 9(1). 10.1186/s13073-017-0472-7 PMC5607840 28934986

[humu24208-bib-0009] Gilman, S. R., Iossifov, I., Levy, D., Ronemus, M., Wigler, M., & Vitkup, D. (2011). Rare de novo variants associated with autism implicate a large functional network of genes involved in formation and function of synapses. Neuron, 70(5), 898–907. 10.1016/j.neuron.2011.05.021 21658583 PMC3607702

[humu24208-bib-0010] Grant, S. G. N. (2019). Synapse diversity and synaptome architecture in human genetic disorders. Human Molecular Genetics, 28, R219–R225. 10.1093/hmg/ddz178 31348488 PMC6872429

[humu24208-bib-0011] Guna, A., & Hegde, R. S. (2018). Transmembrane domain recognition during membrane protein biogenesis and quality control. Current Biology, 28, R498–R511. 10.1016/j.cub.2018.02.004 29689233

[humu24208-bib-0012] Karaca, E., Harel, T., Pehlivan, D., Jhangiani, S. N., Gambin, T., Coban, Akdemir, Z., Gonzaga‐Jauregui, C., Erdin, S., Bayram, Y., Campbell, I. M., Hunter, J. V., Atik, M. M., Van Esch, H., Yuan, B., Wiszniewski, W., Isikay, S., Yesil, G., Yuregir, O. O., … Lupski, J. R. (2015). Genes that affect brain structure and function identified by rare variant analyses of mendelian neurologic disease. Neuron, 88(3), 499–513. 10.1016/j.neuron.2015.09.048 26539891 PMC4824012

[humu24208-bib-0013] Krishnan, A., Zhang, R., Yao, V., Theesfeld, C. L., Wong, A. K., Tadych, A., Volfovsky, N., Packer, A., Lash, A., & Troyanskaya, O. G. (2016). Genome‐wide prediction and functional characterization of the genetic basis of autism spectrum disorder. Nature Neuroscience, 19, 1454–1462. 10.1038/nn.4353 27479844 PMC5803797

[humu24208-bib-0014] Kumar, P., Henikoff, S., & Ng, P. C. (2009). Predicting the effects of coding non‐synonymous variants on protein function using the SIFT algorithm. Nature Protocols, 4, 1073–1081. 10.1038/nprot.2009.86 19561590

[humu24208-bib-0015] Laumonnier, F., Cuthbert, P. C., & Grant, S. G. (2007). The role of neuronal complexes in human X‐linked brain diseases. The American Journal of Human Genetics, 80, 205–220. 10.1086/511441 17236127 PMC1785339

[humu24208-bib-0016] Marshall, C. R., Noor, A., Vincent, J. B., Lionel, A. C., Feuk, L., Skaug, J., Shago, M., Moessner, R., Pinto, D., Ren, Y., Thiruvahindrapduram, B., Fiebig, A., Schreiber, S., Friedman, J., Ketelaars, C. E., Vos, Y. J., Ficicioglu, C., Kirkpatrick, S., Nicolson, R., … Scherer, S. W. (2008). Structural variation of chromosomes in autism spectrum disorder. The American Journal of Human Genetics, 82, 477–488. 10.1016/j.ajhg.2007.12.009 18252227 PMC2426913

[humu24208-bib-0017] Moyses‐Oliveira, M., Yadav, R., Erdin, S., & Talkowski, M. E. (2020). New gene discoveries highlight functional convergence in autism and related neurodevelopmental disorders. Current Opinion in Genetics and Development, 65, 195–206. 10.1016/j.gde.2020.07.001 32846283

[humu24208-bib-0018] Murakami, Y., Imamura, Y., Saito, K., Sakai, D., & Motoyama, J. (2019). Altered kynurenine pathway metabolites in a mouse model of human attention‐deficit hyperactivity/autism spectrum disorders: A potential new biological diagnostic marker. Scientific Reports, 9(1). 10.1038/s41598-019-49781-y PMC6742629 31515500

[humu24208-bib-0019] Nguyen, T. A., Wu, K., Pandey, S., Lehr, A. W., Li, Y., Bemben, M. A., Badger, J. D., 2nd, Lauzon, J. L., Wang, T., Zaghloul, K. A., Thurm, A., Jain, M., Lu, W., & Roche, K. W. (2020). A cluster of autism‐associated variants on X‐linked NLGN4X functionally resemble NLGN4Y. Neuron, 106(5), 759–768. 10.1016/j.neuron.2020.03.008 32243781 PMC7491604

[humu24208-bib-0020] Noor, A., Whibley, A., Marshall, C. R., Gianakopoulos, P. J., Piton, A., Carson, A. R., Orlic‐Milacic, M., Lionel, A. C., Sato, D., Pinto, D., Drmic, I., Noakes, C., Senman, L., Zhang, X., Mo, R., Gauthier, J., Crosbie, J., Pagnamenta, A. T., Munson, J., … Vincent, J. B. (2010). Disruption at the PTCHD1 Locus on Xp22.11 in Autism spectrum disorder and intellectual disability. Science Translational Medicine, 2(49). 10.1126/scitranslmed.3001267 PMC2987731 20844286

[humu24208-bib-0021] Ott, C. M., & Lingappa, V. R. (2002). Integral membrane protein biosynthesis: Why topology is hard to predict. Journal of Cell Science, 115, 2003–2009. 10.1242/jcs.115.10.2003 11973342

[humu24208-bib-0022] Parenti, I., Rabaneda, L. G., Schoen, H., & Novarino, G. (2020). Neurodevelopmental disorders: From genetics to functional pathways. Trends in Neuroscience, 43(8), 608–621. 10.1016/j.tins.2020.05.004 32507511

[humu24208-bib-0023] Pinto, D., Pagnamenta, A. T., Klei, L., Anney, R., Merico, D., Regan, R., Conroy, J., Magalhaes, T. R., Correia, C., Abrahams, B. S., Almeida, J., Bacchelli, E., Bader, G. D., Bailey, A. J., Baird, G., Battaglia, A., Berney, T., Bolshakova, N., Bölte, S., … Betancur, C. (2010). Functional impact of global rare copy number variation in autism spectrum disorders. Nature, 466, 368–372. 10.1038/nature09146 20531469 PMC3021798

[humu24208-bib-0024] Quartier, A., Courraud, J., Thi Ha, T., McGillivray, G., Isidor, B., Rose, K., Drouot, N., Savidan, M. A., Feger, C., Jagline, H., Chelly, J., Shaw, M., Laumonnier, F., Gecz, J., Mandel, J. L., & Piton, A. (2019). Novel mutations in NLGN3 causing autism spectrum disorder and cognitive impairment. Human Mutation, 40, 2021–2032. 10.1002/humu.23836 31184401

[humu24208-bib-0025] Remerand, G., Boespflug‐Tanguy, O., Tonduti, D., Touraine, R., Rodriguez, D., Curie, A., Perreton, N., Des Portes, V., & Sarret, C., RMLX/AHDS Study Group . (2019). Expanding the phenotypic spectrum of Allan‐Herndon‐Dudley syndrome in patients with SLC16A2 mutations. Developmental Medicine and Child Neurology, 61, 1439–1447. 10.1111/dmcn.14332 31410843

[humu24208-bib-0026] Salgado, D., Desvignes, J. P., Rai, G., Blanchard, A., Miltgen, M., Pinard, A., Lévy, N., Collod‐Béroud, G., & Béroud, C. (2016). UMD‐predictor: A high‐throughput sequencing compliant system for pathogenicity prediction of any human cDNA substitution. Human Mutation, 37, 439–446. 10.1002/humu.22965 26842889 PMC5067603

[humu24208-bib-0027] Sievers, F., Wilm, A., Dineen, D., Gibson, T. J., Karplus, K., Li, W., Lopez, R., McWilliam, H., Remmert, M., Söding, J., Thompson, J. D., & Higgins, D. G. (2011). Fast, scalable generation of high‐quality protein multiple sequence alignments using Clustal Omega. Molecular Systems Biology, 7, 539. 10.1038/msb.2011.75 21988835 PMC3261699

[humu24208-bib-0028] Tora, D., Gomez, A. M., Michaud, J. F., Yam, P. T., Charron, F., & Scheiffele, P. (2017). Cellular functions of the autism risk factor PTCHD1 in mice. Journal of Neuroscience, 37, 11993–12005. 10.1523/JNEUROSCI.1393-17.2017 29118110 PMC6596831

[humu24208-bib-0029] Torrico, B., Fernàndez‐Castillo, N., Hervás, A., Milà, M., Salgado, M., Rueda, I., Buitelaar, J. K., Rommelse, N., Oerlemans, A. M., Bralten, J., Freitag, C. M., Reif, A., Battaglia, A., Mazzone, L., Maestrini, E., Cormand, B., & Toma, C. (2015). Contribution of common and rare variants of the PTCHD1 gene to autism spectrum disorders and intellectual disability. European Journal of Human Genetics, 23, 1694–1701. 10.1038/ejhg.2015.37 25782667 PMC4795195

[humu24208-bib-0030] Ung, D. C., Iacono, G., Méziane, H., Blanchard, E., Papon, M. A., Selten, M., van Rhijn, J. R., Montjean, R., Rucci, J., Martin, S., Fleet, A., Birling, M. C., Marouillat, S., Roepman, R., Selloum, M., Lux, A., Thépault, R. A., Hamel, P., Mittal, K., … Laumonnier, F. (2018). Ptchd1 deficiency induces excitatory synaptic and cognitive dysfunctions in mouse. Molecular Psychiatry, 23, 1356–1367. 10.1038/mp.2017.39 28416808 PMC5984103

[humu24208-bib-0031] Wells, M. F., Wimmer, R. D., Schmitt, L. I., Feng, G., & Halassa, M. M. (2016). Thalamic reticular impairment underlies attention deficit in Ptchd1(Y/‐) mice. Nature, 532, 58–63. 10.1038/nature17427 27007844 PMC4875756

[humu24208-bib-0032] Whibley, A. C., Plagnol, V., Tarpey, P. S., Abidi, F., Fullston, T., Choma, M. K., Boucher, C. A., Shepherd, L., Willatt, L., Parkin, G., Smith, R., Futreal, P. A., Shaw, M., Boyle, J., Licata, A., Skinner, C., Stevenson, R. E., Turner, G., Field, M., … Raymond, F. L. (2010). Fine‐scale survey of X chromosome copy number variants and indels underlying intellectual disability. The American Journal of Human Genetics, 87, 173–188. 10.1016/j.ajhg.2010.06.017 20655035 PMC2917707

[humu24208-bib-0033] Zhang‐James, Y., Vaudelv, M., Mjaavattenv, O., Bervenv, F. S., Haavikv, J., & Faraone, S. V. (2019). Effect of disease‐associated SLC9A9 mutations on protein‐protein interaction networks: Implications for molecular mechanisms for ADHD and autism. ADHD Attention Deficit Hyperactivity Disorders, 11, 91–105. 10.1007/s12402-018-0281-x 30927234

