# Supplementary material for: Novel missense mutations in PTCHD1 alter its plasma membrane subcellular localization and cause intellectual disability and autism spectrum disorder
Source: Hum Mutat. 2021 May 3;42(7):848–61. doi: 10.1002/humu.24208 (PMC8359977; doi:10.1002/humu.24208)
Supplement: Supplementary file 1 — Supplementary information. [file HUMU-42-848-s001.docx]

**Supplementary Figure S1. Colocalization analysis of PTCHD1-GFP proteins within the proteasome in HEK 293T cells**.

(A) Representative confocal microscopy images of HEK293T cell lines transfected with GFP, PTCHD1 expression plasmids, GFP-tagged wild-type (WT) or variants of PTCHD1, and stained with anti-GFP antibody (PTCHD1-GFP, green), anti-PSMB5 antibody (Proteasome P20S, red) and DAPI (nucleus, blue). Scale bar, 20µm. (B) Manders’ coefficient of co-localization calculating the percentage of PTCHD1-GFP WT or variants overlapping PSMB5 staining. Kruskal-Wallis test with Dunn's multiple comparisons tests were made to compare each Ptchd1 variant to the WT. Normality of each condition was measured by d’Agostino-Pearson normality test. n= 3 independent transfections with 7-10 images analyzed per condition. ns: not significant; *p<0.05; **p<0,01; error bars represent SEM. Scale bar, 20µm.

**Supplementary Table S1.** **List and sequence of the back-to-back primers used for the site-directed mutagenesis.** The lower case letter in the sequence indicates the nucleotide variant. Designed using NEBaseChanger.neb.com website.
